# A False Case of Clozapine-Resistant Schizophrenia

**DOI:** 10.1155/2010/534027

**Published:** 2010-03-11

**Authors:** J. P. Maia-de-Oliveira, J. P. Pinto, V. Alexandre, J. P. Machado-de-Sousa, S. L. Morais, C. Chaves, A. C. Sakamoto, A. W. Zuardi, J. A. S. Crippa, J. E. Hallak

**Affiliations:** Department of Neuroscience and Behavioral Sciences, Ribeirao Preto Medical School, University of São Paulo (USP), Hospital das Clinicas, TerceiroAndar, AvenidaBandeirantes Campus Universitario, 3900, Ribeirao Preto, SP 14049-900, Brazil

## Abstract

One of the subjects that most concerns physicians is treatment-resistance. About 30%–60% of schizophrenia patients do not respond adequately to antipsychotic treatment and are known as refractory schizophrenia patients. Clozapine has been the drug of choice in such cases. However, approximately 30% of them do not respond to clozapine either. Here, we describe a patient with an initial diagnosis of refractory schizophrenia who had a history of dramatic aggressiveness. However, in this case, “refractoriness” was a wrong diagnosis. A case of psychosis secondary to epilepsy had been treated as schizophrenia for almost 20 years. Reports like this one are important because they remind us of how a thorough investigation can lead to the correct diagnosis and improve the patient's prognosis.

## 1. Introduction

About 30%–60% of schizophrenia patients do not respond adequately to antipsychotic treatment and are known as refractory schizophrenia patients. Refractory schizophrenia criteria were established based on Kane's studies with clozapine and modified by the *The International Psychopharmacology Algorithm Project* (IPAP): (1) no period of good functioning in the previous 5 years, (2) prior nonresponse to at least 2 antipsychotic drugs of two different chemical classes for at least 4–6 weeks each at doses ≥ 400 mg equivalents of chlorpromazine or 5 mg/day of risperidone and (3) moderate to severe psychopathology, especially positive symptoms. Clozapine has been the drug of choice in such cases. However, approximately 30% of them do not respond to clozapine either [1, 2]. 

Sometimes we are forced to review our diagnostic steps. This is the case when a patient's psychiatric disorder does not respond to treatments that were supposed to work. One relevant step in the reassessment of the diagnosis is the exclusion of an underlying medical condition. According to the *Diagnostic and Statistical Manual of Mental Disorders* (DSM-IV-TR), the diagnosis of any psychiatric disorder requires a thorough investigation and exclusion of possible general medical causes that could better explain the symptoms. Serious medical conditions may be overlooked when this recommendation is disregarded [3]. 

In this case report, refractoriness was a wrong diagnosis. A case of psychosis secondary to epilepsy had been treated as schizophrenia for almost 20 years.

## 2. Case Presentation

Mr A, 38 years old, was referred for expert evaluation in 2005. He had a normal development until he was eight, when he started to present irritability and soliloquies. At 19, he began his psychiatric treatment due to agitation, visual and auditory hallucinations, and delusions of persecution. He was 25 when he beat his mother and was hospitalized for the first time.

The patient had already been treated with haloperidol 30 mg/day, chlorpromazine 600 mg/day, and risperidone 6 mg/day.

He had no history of putative symptoms of epilepsy, such as olfactory hallucinations or seizures. He had no history of traumatic head injury or family history of seizures. However, his 45-year-old brother had a stroke two years ago and one of his cousins had a diagnosis of schizophrenia.

Mr A had been physically restrained at a traditional psychiatric hospital for most of the previous year due to recurrent episodic aggressiveness. The psychiatrist described: “The patient was hospitalized several times in the last years due to his psychosis and aggressiveness. Few days after discharge, he was brought back to the hospital after breaking everything at home”; his brother added: “Many times my family needed to have medical assistance as a consequence of his violence.”

Blood tests, serology (including VDRL and HIV), screening for drugs, EEG, Cerebral Tomography, and MRI showed no abnormalities. Thus, following schizophrenia guidelines, a clinical trial with olanzapine (a second-generation antipsychotic) was suggested [4].

After almost one year, in November 2006, he was referred to our inpatient unit with a diagnosis of clozapine-resistant schizophrenia. He had been on clozapine 400 mg/day for eight months after failed trials with olanzapine (20 mg/day) and quetiapine (800 mg/day).

In his first weeks of hospitalization, the dose of clozapine was increased up to 600 mg/day. However, the patient continued presenting episodes of agitation and aggressiveness. He said that he was haunted by his dead father. At these moments, he would not respond to verbal interaction, requiring the prescription of physical contention and parentheral sedative medications.

The patient would not attend the therapeutical activities and had to be watched full time.

Sometimes he alleged he did not remember what happened. Sometimes, he tried to justify himself: “I'm sick, I can't control myself. I need to be tied up not to beat anyone.”

One day, after an episode in which he shouted out obscenities and tried to punch a nurse, the patient presented dizziness and sleepiness. Because of this peculiar pattern of symptoms and his unresponsiveness to clozapine, we insisted on a 48-hour Video-EEG monitoring at our Epilepsy Center.

**Figure 1:**
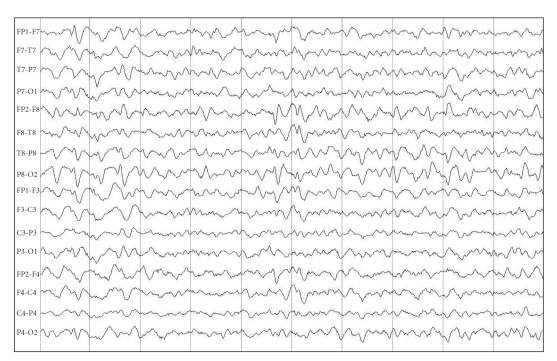
EEG before valproate therapy showing bilateral sharp wave discharges.

This new investigation revealed bilateral temporal paroxysm (Figure 1). Therefore, we changed our conduct by starting valproate and gradually discontinuing clozapine.

**Figure 2:**
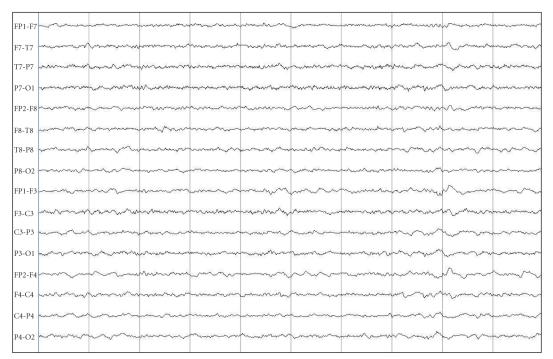
EEG after valproate therapy showing improvement of cerebral rhythms.

The patient presented remission of his aggressiveness and psychosis, started to attend the therapeutical activities, and improved social interaction and self-care. Another Video-EEG revealed improvement in the pattern observed in the previous exam (Figure 2). He was discharged using valproate 2000 mg/day (serum level: 88 mg/L). When last contacted, in March 2009, the patient was much better.

## 3. Discussion

Epilepsy is commonly associated with psychosis. The seizures are range variable and a routine EEG can be normal. Several studies have suggested an association between these two conditions. The frequency of psychosis has been reported to be higher in patients with epilepsy compared with the general population [5, 6]. 

Discrimination based on the chronologic relation between the psychotic episodes and seizures has been widely used and includes: (a) interictal psychosis, in which the presence of psychotic episodes is not temporally related to the occurrence of seizures; (b) postictal psychosis, characterized by an increased number of seizures followed by a period of lucidity and subsequent psychotic symptoms; and (c) ictal psychosis, in which psychotic symptoms occur in association with ictal discharges on EEG. Also, there are other kinds of episodic symptoms that may mimic psychosis, including nonconvulsive status epilepticus, postictal delirium, and peri-ictal aggressive behavior [5]. 

Unfortunately, there is insufficient biologic evidence to support these chronology-based classifications of psychoses as distinct clinical entities. Some clinical features are common to them and, over time, in some patients who initially manifested postictal psychosis, interictal psychosis develops. Actually, there is no consensus as to how those cases should be classified [7–9]. However, we believe that the most important message of this manuscript is that, when evaluating a patient with psychiatric symptoms, it is essential to bear in mind the possibility of a general medical condition. This differential diagnosis cannot be forgotten even when in the absence of exuberant neurological signs and symptoms.
